# A 3D Follow-Up Study of Cranial Asymmetry from Early Infancy to Toddler Age after Preterm versus Term Birth

**DOI:** 10.3390/jcm8101665

**Published:** 2019-10-11

**Authors:** Anniina M. Launonen, Henri Aarnivala, Panagiotis Kyteas, Ville Vuollo, Tuomo Heikkinen, Chung H. Kau, Pertti Pirttiniemi, Virpi Harila, A. Marita Valkama

**Affiliations:** 1 Department of Oral Development and Orthodontics, Oulu University Hospital, Oulu, Finland; ville.vuollo@oulu.fi (V.V.); tuomo.heikkinen@oulu.fi (T.H.); pertti.pirttiniemi@oulu.fi (P.P.); virpi.harila@oulu.fi (V.H.); 2 Department of Oral Development and Orthodontics, Unit of Oral Health Sciences, Faculty of Medicine, University of Oulu, Oulu, Finland; 3 Medical Research Center Oulu, Oulu, Finland; henri.aarnivala@student.oulu.fi (H.A.); marita.valkama@ppshp.fi (A.M.V.); 4 Department of Children and Adolescents, Oulu University Hospital, Oulu, Finland; 5 PEDEGO Research Group, University of Oulu, Oulu, Finland; 6 Department of Orthodontics, University of Alabama, Birmingham, AL 35294-0007, USA; panoskyt@uab.edu (P.K.); ckau@uab.edu (C.H.K.)

**Keywords:** preterm, 3D-imaging, deformational plagiocephaly, cranial growth, oblique cranial length ratio, cephalic index, flatness score, weighted asymmetry score

## Abstract

Preterm infants are at higher risk for both symmetrical and asymmetrical head molding. This study involved 3D stereophotogrammetry to assess the cranial growth, molding, and incidence of deformational plagiocephaly (DP) in preterm children compared to term born children. Thirty-four preterm infants and 34 term born controls were enrolled in this study from Oulu University Hospital, Finland. Three-dimensional head images were obtained at the age of 2–4 months (T1), 5–7 months (T2), 11–13 months (T3), and 2.5–3 years (T4) from the term equivalent age (TEA). There was no statistically significant difference in oblique cranial length ratio (OCLR), cephalic index (CI), or weighted asymmetry score (wAS) between the two groups. Occipital flattening, defined by flatness score (FS) was statistically significantly greater in the preterm group than in the term group at T1–T4 (*p* < 0.05). In both groups, OCLR improved gradually over time. There were no instances, in either group, of severe DP and no moderate DP after T2. Results indicate that DP affects preterm and full-term children almost equally during the first three years of life, and cranial asymmetry resolves at a similar rate in both preterm and term groups after three months of corrected age. Preterm infants present with more occipital flattening than full-term children.

## 1. Introduction

Deformational plagiocephaly (DP) refers to an asymmetrical head shape resulting from environmental forces on the infant’s cranium. Since 1992, the American Academy of Pediatrics (AAP) has recommended that infants sleep in a supine position to prevent sudden infant death syndrome (SIDS). After that, the reported prevalence of DP has risen as high as 46.6% in Canada [1,2,3]. However, the reported prevalence rates have a wide range, as the prevalence is affected by the evaluation age of the infants, the diagnostic methods used, the decade when the research was performed, and the prevention and follow-up aspects offered by local health professionals [4,5,6,7,8,9,10,11].

The most common visible features in DP are one-sided occipital flattening, forward displacement of the same-sided ear and malar complex, same-sided frontal bossing, and opposite side occipital bossing [11,12,13,14,15,16]. Frontal DP is uncommon [17]. A hairless bald area on the flattened side of the head, a preferred head position, and a restricted cervical motion are common associated findings.

Because the skullcap of preterm infants (born at a gestational age less than 37 weeks) is very thin and malleable during the early months of life, preterm infants are, according to the literature, at a higher risk for both symmetrical and asymmetrical head molding. The more preterm the infant is, the longer the time from birth to full control of head position; hence, the most immature preterm infants are the most prone to external cranial molding. Cranial narrowing, or dolichocephaly, is commonly described as a typical cranial appearance of very preterm infants, especially at term equivalent age (TEA) [18,19]. Additionally, preterm infants go through a long period of hospital care before their discharge home, and if no attention is paid to varying the head position from early on in the neonatal intensive care unit (NICU), preterm infants frequently develop DP [19,20,21]. If preventive and proactive positioning advice in infant care and handling are provided to the guardians early enough, at least the most severe forms of DP can be prevented in term or near-term newborns infants [3,7,22,23].

The aim of this study was to use 3D stereophotogrammetry to assess cranial molding with a special emphasis on the incidence of plagiocephaly in preterm children in comparison to term born children in early childhood.

## 2. Materials and Methods

### 2.1. Study Population and Study Design

This prospective case-control study was conducted in the Department of Children and Adolescents, Oulu University Hospital, and the Unit of Oral Health Sciences, University of Oulu, Finland. Thirty-four healthy preterm infants (born before 37 completed gestation weeks), and 34 healthy term born controls (born after 37 completed gestation weeks), were enrolled into this follow-up study. There were 11 females and 23 (67.6%) males in each group. The control group was randomly selected from a previously collected nonintervention cohort [8] by computer-based random selection and matched to gender. All participants were born on preselected dates between the years 2012 and 2015 in Oulu University Hospital and entered into the study before their discharge home after birth. Infants were considered eligible if they had no cheilopalatoschisis, craniosynostosis, or dysmorphic features and if they resided within the Oulu region.

Approval for the study was obtained from the ethics committee of the Northern Ostrobothnia Hospital District (Oulu University Hospital; EETTMK 27/2011), and the study complies with the Declaration of Helsinki. The parents of participants authorized study entry by written informed consent. The study was registered with the National Clinical Trials register (NCT02283229).

To reliably compare preterm subjects to term born controls, their ages were computed from the expected date of birth, the term equivalent age (TEA). Subjects attended the study at predetermined time points from the TEA up to the age of 3 years targeted as follows: 2–4 months (T1), 5–7 months (T2), 11–13 months (T3), and 2.5–3 years (T4). At each visit, 3D images of the head were obtained using a 3dMD head 5-pod camera system (3dMD, Atlanta, Georgia, USA). Height, weight, and head circumference measurements were obtained at birth and T1–T4 visits.

### 2.2. 3D Image Analysis

The 3D images were processed and analyzed with the Rapidform 2006 (Geomagic, Rock Hill, SC, USA) 3D software system using custom macros written with Visual Basic for Applications (VBA). More complex mathematical analyses were performed with MATLAB R2014b (MathWorks, Natick, MS, USA).

**Figure 1:**
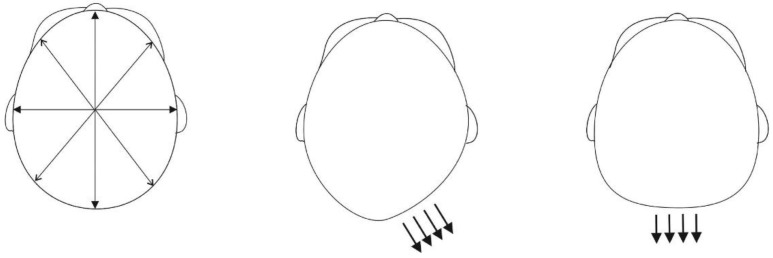
Oblique cranial length ratio (OCLR, left figure) = ratio of the longer and shorter transcranial diagonal, measured at a 40° angle to the midline: (longer diagonal/shorter diagonal) × 100%. Cephalic index (CI, left figure) = ratio between the width and length of the head (transversal diameter/sagittal diameter) × 100%. On a flat surface, surface normal vectors have parallel direction leading to a local maximum in the density function of direction angles. Weighted asymmetry score (wAS, middle figure) = an asymmetrical local maximum in the density function, flatness score (FS, right figure) = a symmetrical local maximum in the density function. FS and wAS are calculated by integrating the kernel density estimation function and in wAS, the integrand is multiplied by weight coefficient based on the most posterior point on the occiput.

The point-to-point variables oblique cranial length ratio (OCLR) and cephalic index (CI), as well as the 3D surface variables, weighted asymmetry score (wAS), and flatness score (FS) based on surface normal vector distribution, were measured from the 3D images [24] (Figure 1).

The diagnosis of DP was based on OCLR, with 104% as the cut-off value for DP, which has been described as the most optimal and clinically relevant cut-off point [4,25]. The severity of DP was also categorized based on OCLR: OCLR 104–107.9% indicates mild; OCLR 108–111.9% indicates moderate; and OCLR ≥ 112% indicates severe DP [5,7].

### 2.3. Statistical Analysis

The normality was assessed using the Shapiro–Wilk normality test. The independent samples *t*-test was used to compare maternal age, gestational age, weight, length, and head circumference between preterm and full-term groups for normally distributed data, and the Mann–Whitney test was used for non-normally distributed data. For outcome variables OCLR, CI, wAS, and FS, the normal distribution of the data could not be verified, so the Mann–Whitney test was used to compare variables between preterm and full-term groups and further between the following subgroups: nonsingletons and singletons, and boys and girls. To compare variables between the subgroups of very preterm (VPT), moderate preterm (MPT), and late preterm (LPT) infants, the Kruskal–Wallis test was used, and Bonferroni corrections were used for post-hoc analyses. The level of significance was set at *p* < 0.05. All statistical work and data analyses were conducted using IBM SPSS Statistics version 25.

## 3. Results

Of the 34 preterm and 34 term born infants who participated in this study, two moved away from the area and were unable to visit at T3 and T4. Two preterm infants were unable to show up at T3 but were able to return at T4. One term born control participant refused to put a stocking cap on at T4, so the images could not be analyzed because of the hair. The mean (SD) corrected ages of preterm versus term born participants were in T1, 3.0 (0.8) versus 3.6 (1.0) months (*p* = 0.012); in T2, 6.5 (1.2) versus 6.8 (1.0) months; in T3, 12.9 (1.2) versus 13.3 (0.7) months; and in T4, 3.3 (0.1) versus 3.3 (0.1) years. There were no statistically significant differences in the mean ages of the groups at timepoints T2–T4.

There were 11 female and 23 (67.6%) male subjects in each group. All control cases and 14 (41.2%) of the preterm cases were born as singletons. Ten cases of the preterm group were very preterm (VPT, born before 32 completed gestation weeks); 13 were moderate preterm (MPT, born before 34 but more than 32 gestation weeks); and 11 were late preterm (LPT, born between 34 and 37 gestation weeks). Subject characteristics and common growth data are shown in Table 1.

**Figure 2:**
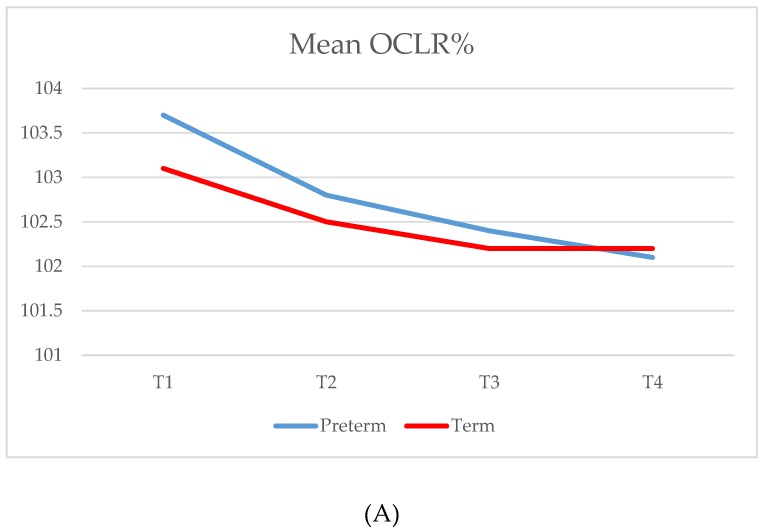

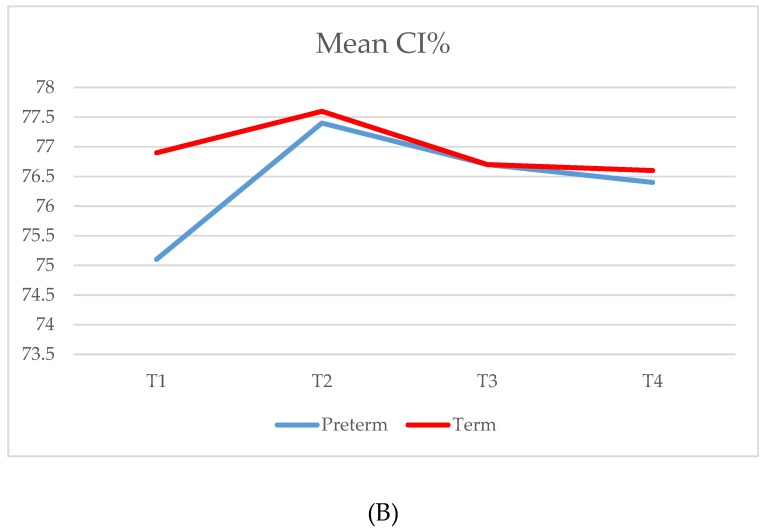
Mean OCLR% (**A**) and CI% (**B**) at the study time points (T1–T4) for preterm (PT) and term (T) groups.

As shown in Table 2, no statistically significant difference in OCLR between preterm and full-term children was noted. In both groups, OCLR improved gradually over time (Figure 2). Equally, the number of DP in both groups decreased over time from T1 to T4 (Table 3). There were no severe DP in either group at any time, and there were no instances of moderate DP after T2. Between the two groups, there was no statistically significant difference in the mean CI. However, the mean FS, indicating occipital flatness, was statistically significantly greater in the preterm group than in the term group in every time point T1–T3 (Table 2).

There were no statistically significant differences in the mean values of OCLR, CI, wAS, or FS between the genders.

Table 4 describes the cranial parameters between the subgroups of VPT, MPT, and LPT infants. CI was smaller in the VPT group compared to the LPT group at T1 and T4. The median value of OCLR was significantly greater in the subgroup of LPT infants compared to MPT infants at T3–T4 (Table 4).

## 4. Discussion

This follow-up study characterizes the cranial growth features and the course of cranial symmetry and shape in preterm versus term born infants from early infancy to toddler age in the Finnish population. Our study is the first known longitudinal study using 3D imaging to examine the development of cranial shape and the natural course of DP in preterm children compared with full-term children up to the age of three years.

Data detailing the pattern of head growth of infants in this study were in accordance with previous data from large cohorts [26,27], and there were no statistically significant differences between groups in the weight, length, or head circumference after T1, where preterm infants were younger than term controls. Among both preterm and full-term children, the course of cranial asymmetry was equally favorable between T1 and T4; both the highest mean value of OCLR and the highest point prevalence for DP were observed at T1, with up to one-third of the children in both groups qualifying as having DP. Subsequently, the incidence of DP gradually diminished equally in both groups. The course of DP in full-term children follows a similar pattern worldwide, as our results are similar to other reports in various populations [5,28,29].

Understandably, the risk for developing DP is highest during the first months of life, when infants lie in the supine position most of the time and often have a preferential side for lateral head orientation [30]. Once infants are able to change the head position independently, the impact of external forces and pressure on the occiput rapidly decrease [5]. The head orientation profile of healthy full-term infants normally changes from lateral head orientation to midline orientation in three to nine weeks after birth, and a delay in that maturation seems to associate with DP [31]. Accordingly, head turn preference at TEA in preterm infants has been associated with DP, but also with the severity of medical comorbidities and poor neurobehavioral performance [19,32,33,34].

Somewhat surprisingly, LPT infants had considerably more cranial asymmetry in terms of both OCLR and wAS than earlier born preterm infants. In a cross-sectional study of preterm infants in TEA, Ifflaender et al. reported quite conversely that very preterm infants had statistically significantly more plagiocephaly compared to late preterm and term born infants [19]. Our study results are not, however, fully comparable with Ifflaender et al. because the observation period in our study started when the infants were three months old. Nevertheless, the prevalence of DP among VPT and MPT infants is far lower in this population at the ages of three and six months than has been reported in the literature [33,35]. These findings are likely explained by the fact that throughout their stay in the NICU, nursing staff members pay attention to varying each infant’s position, usually changing it every 3–4 h according to treatments. Parents are also able to get the infants to kangaroo care on a daily basis. Additionally, parents get repeated guidance on infant handling and positioning from the nursing staff and from a specialized physiotherapist before discharge.

After discharge, all infants follow the regular Finnish well child visit schedule, including visits to a primary healthcare nurse on a monthly to bimonthly basis as well as to a primary healthcare physician at approximately two, four, and eight months of age. VPT infants additionally visit a pediatrician and a physiotherapist at three-month intervals. Advice on positioning and handling may be provided at any of these visits, and whenever necessary, physiotherapy may be commenced. None of the infants in this study received helmet therapy. It can be inferred that the systematic follow-up of the development of both preterm and term born infants in Finland may have a great impact on the prevention of both DP and particularly more severe forms of the condition.

Although the incidence of DP decreased throughout the whole observation period both in preterm and term groups, there was a notable amount of cranial asymmetry still present at the age of three years. The amount of residual cranial asymmetry at the age of three in this study is analogous with an earlier European study [5]. It is known that DP may take a long time to resolve [28,36], and sometimes it may be permanent, as Ruby et al. report the prevalence of deformational cranial abnormalities to be 2% in teens born after the ‘Back to Sleep’ campaign [36]. Furthermore, although DP is usually transient, it may be associated with delayed motor development or torticollis [11,13,33,34]. DP also associates with a rotation of the cranial base and the anterior displacement of the articular fossa on the affected side, which is hypothesized to cause facial asymmetry [37,38,39]. To prevent and to detect these later consequences of DP, a systematic follow-up combined with proactive advice for infant handling, especially during NICU care, might be useful, particularly for preterm infants.

The early-born preterm infants in this study had a more dolichocephalic skull shape than late-born preterm infants. A similar finding was reported by Ifflaender et al. [19], who also observed that among preterm children, CI increased from discharge to the age of three months and further to the age of six months [35]. In our study population, a similar increase in CI from T1 to T2 in every subgroup was observed, whereafter there was a slightly decreasing trend toward T4 (Figure 2). Generally, both Finnish preterm and term born infants and toddlers have more dolichocephalic skull shapes than what has been reported from other populations [26,29,35,40], which might be either purely ethnic or a generational phenomenon [14].

Despite being more dolichocephalic, the preterm children had more symmetrical occipital flatness than term ones, as indicated by FS values (Figure 1). Hence, it appears that the particularly malleable skullcap of preterms not only tends to narrow bilaterally but also flattens occipitally more than the cranium of term infants. This finding further highlights the importance of preventive and positioning advice at an early age [7], especially with preterm infants, in order to direct the growth of the cranium to the symmetric direction.

The strengths of this study include its prospective setting; a long follow-up time up to three years of age; its randomly-selected, full-term control peers; and the use of 3D imaging, which brings systematism, consistency, and objectivity to analysis [11,40,41,42]. However, the use of the stereophotogrammetry method requires subjects to support their head in the upright position during imaging. Therefore stereophotogrammetry is not the proper method for studying infants at TEA or other time points in early life. For this reason, the time interval to follow cranial molding from three months to three years was chosen, which might be considered a weakness. However, this period includes the peak prevalence of DP, and during this period, the majority of spontaneous resolving of cranial asymmetry is expected to happen [8,43]. Also, the differences in cranial shape and symmetry between preterm and full-term born children in TEA has been documented earlier [19].

The small number of subjects in the preterm subgroups is a limitation of this study. Nevertheless, the sample size was considered adequate for comparing cranial molding and the course of cranial asymmetry between preterm and full-term children, which was the main aim of this study. A final limitation is that unlike the case of plagiocephaly, there are no established cut-off values for dolichocephaly or brachycephaly, and cut-off values used in previous studies vary widely [5,19,44,45]. Thus, comparing the incidence of dolichocephaly or brachycephaly is problematic between different studies and populations. Because of that, we decided to report and discuss only the absolute values for the cephalic index.

Even though it appears that parental guidance and the attention paid to infant positioning in specialized care might prevent plagiocephaly, no reliable conclusions cannot be drawn from this study as it was not designed to test this hypothesis. Therefore, future research should focus on the effect of DP prevention among preterm born infants in NICU. Also, there is still a lack of information about the later consequences of DP, especially its effects on the facial asymmetry or occlusal disorders later in life.

## 5. Conclusions

In this study, DP was found to affect preterm and full-term children almost equally during the first three years of life, and cranial asymmetry resolved at a similar rate in both preterm and term born children after three months of corrected age. The average cranial shape among Finnish children is relatively dolichocephalic. Very preterm born infants have a more dolichocephalic skull shape than late preterm, but despite this, preterm infants present with more occipital flattening than full-term children.

## Figures and Tables

**Table 1:** Characteristics of study subjects and growth data at birth and at all time points (T1–T4), presented as mean (SD), median, and range values.

Characteristic	Preterm			Term			
	Mean (SD)	Median	Range	Mean (SD)	Median	Range	*p*
Maternal Age, years	31.3 (5.7)	31.5	26.8–35.0	30.9 (5.4)	30.5	26.8–36.0	0.779 ^1^
Gestational Age, weeks	32.7 (3.2)	33.3	31.1–35.3	40.0 (1.2)	40.0	39.1–41.0	0.000 ^2^
Birth:							
Weight, g	2006.5 (712.3)	1887.5	1460.0–2645.0	3528.5 (361.7)	3470.0	3215.0–3745.0	0.000 ^2^
Length, cm	42.0 (5.0)	42.5	38.8–46.3	50.5 (1.8)	50.25	49.0–52.0	0.000 ^1^
Head Circumference, cm	30.6 (3.2)	31.5	28.9–32.6	34.9 (1.3)	34.5	34.0–35.6	0.000 ^2^
T1:							
Weight, kg	5.9 (1.0)	5.93	5.1–6.3	6.4 (9.7)	6.4	5.7–7.9	0.035 ^1^
Length, cm	59.2 (2.9)	58.6	57.4–61.0	61.8 (3.3)	62.25	59.3–64.6	0.001 ^1^
Head Circumference, cm	41.1 (1.8)	40.85	39.5–42.6	41.7 (1.7)	41.45	40.7–43.0	0.145 ^1^
T2:							
Weight, kg	7.9 (1.1)	7.8	7.1–8.8	8.0 (1.0)	8.1	7.3–8.5	0.739 ^1^
Length, cm	67.2 (2.7)	67.0	65.7–68.2	67.9 (2.6)	67.9	65.6–70.2	0.263 ^1^
Head Circumference, cm	44.4 (1.4)	44.2	43.6–45.4	44.5 (1.6)	44.5	43.4–45.8	0.876 ^1^
T3:							
Weight, kg	9.9 (1.4)	9.77	8.8–10.5	9.9 (1.2)	9.9	9.0–10.4	0.971 ^1^
Length, cm	75.4 (2.9)	75.1	73.5–77.2	76.9 (4.2)	76.5	74.0–78.5	0.170 ^2^
Head Circumference, cm	47.6 (1.1)	47.6	46.8–48.2	47.4 (1.5)	47.3	46.3–48.6	0.634 ^1^
T4:							
Weight, kg,	14.4 (1.8)	14.05	13.0–15.6	14.6 (1.6)	14.75	13.7–15.1	0.397 ^2^
Length, cm	94.4 (3.8)	94.2	91.2–95.5	95.5 (4.5)	95.85	93.0–98.4	0.277 ^1^
Head Circumference, cm	50.8 (1.2)	51.0	49.7–51.9	51.1 (1.5)	50.9	50.0–52.4	0.442 ^1^

^1^ The Independent samples *t*-test was used, ^2^ The Mann–Whitney test was used.

**Table 2:** The mean (SD), median, and range values for OCLR, CI, wAS, and FS at the study time points (T1–T4) for preterm (PT) and term (T) groups.

Parameters at T1–T4 (PT/T, Numbers)	Preterm			Term			Mann-Whitney
	Mean (SD)	Median	Range	Mean (SD)	Median	Range	*p*
OCLR %							
T1 (34/34)	103.7 (2.8)	103.1	101.1–105.8	103.1 (2.3)	102.5	101.1–104.6	0.425
T2 (32/34)	102.8 (2.3)	102.2	101.2–103.7	102.5 (2.3)	101.6	100.7–104.0	0.330
T3 (30/34)	102.4 (2.0)	101.8	100.9–103.5	102.2 (1.9)	101.6	100.7–103.3	0.778
T4 (32/33)	102.1 (1.4)	101.7	101.0–103.0	102.2 (1.7)	101.9	100.9–103.3	0.958
CI %							
T1 (34/34)	75.1 (5.4)	73.2	71.4–78.7	76.9 (3.5)	77.1	73.6–79.3	0.057
T2 (32/34)	77.4 (6.1)	75.5	73.1–80.5	77.6 (3.8)	77.4	75.5–80.7	0.293
T3 (30/34)	76.7 (5.7)	75.6	72.8–80.0	76.7 (3.3)	76.7	75.1–78.5	0.484
T4 (32/33)	76.4 (4.6)	76.0	73.2–78.9	76.6 (3.1)	76.9	73.9–78.0	0.529
wAS							
T1 (34/34)	30.7 (36.1)	19.5	3.8–45.4	32.7 (67.5)	14.2	7.6–31.6	0.990
T2 (32/34)	19.5 (24.7)	8.6	2.9–24.3	23.9 (36.1)	9.1	2.8–28.4	0.797
T3 (30/34)	15.0 (15.1)	9.3	4.2–21.0	22.1 (32.7)	10.2	3.5–29.5	0.788
T4 (32/33)	17.6 (21.7)	11.3	3.1–24.9	29.0 (34.8)	15.6	6.5–34.0	0.160
FS							
T1 (34/34)	0.212 (0.019)	0.211	0.198–0.225	0.203 (0.015)	0.199	0.194–0.21	0.030
T2 (32/34)	0.216 (0.022)	0.218	0.196–0.229	0.201 (0.011)	0.200	0.194–0.209	0.003
T3 (30/34)	0.206 (0.018)	0.204	0.191–0.218	0.196 (0.01)	0.194	0.188–0.202	0.037
T4 (32/33)	0.208 (0.016)	0.205	0.195–0.224	0.201 (0.011)	0.200	0.195–0.204	0.081

**Table 3:** Number (N (%)) of preterm and term born subjects with severity levels of deformational plagiocephaly (DP) at timepoints T1–T4.

Timepoints	Severity	Preterm	Term
		*n* = 34	*n* = 34
T1 Plagiocephaly	Total	13 (38.2%)	11 (32.4%)
	Mild	10 (29.4%)	10 (29.4%)
	Moderate	3 (8.8%)	1 (2.9%)
	Severe	0	0
T2 Plagiocephaly	Total	7 (21.9%)	8 (23.5%)
	Mild	5 (15.6%)	7 (20.6%)
	Moderate	2 (6.3%)	1 (2.9%)
	Severe	0	0
T3 Plagiocephaly	Total	6 (20.0%)	6 (17.6%)
	Mild	6 (20.0%)	6 (17.6%)
	Moderate	0	0
	Severe	0	0
T4 Plagiocephaly	Total	4 (12.5%)	5 (15.2%)
	Mild	4 (12.5%)	5 (15.2%)
	Moderate	0	0
	Severe	0	0

**Table 4:** The median and range values for OCLR, CI, wAS, and FS at the study time points (T1–T4) in preterm subgroups.

Parameters at Timepoints								Kruskal-Wallis	Post-Hoc Analysis		
T1–T4	n	VPT		MPT		LPT			MPT-VPT	LPT-VPT	LPT-MPT
		Median	Range	Median	Range	Median	Range	*p*	*p*	*p*	*p*
OCLR %											
T1	10/13/11	102.3	101.1–104.2	102.1	101.0–105.3	105.8	102.5–108.7	0.106	–	-	-
T2	8/13/11	102.0	101.3–104.6	101.7	100.7–103.1	103.5	101.3–106.7	0.262	–	-	-
T3	8/13/9	102.0	101.2–104.0	101.2	100.3–102.0	102.9	102.1–106.3	0.032	0.511	0.878	0.028
T4	10/12/10	101.6	101.1–102.9	101.1	100.7–102.5	102.6	101.7–104.7	0.046	1.000	0.199	0.051
CI %											
T1	10/13/11	70.0	68.4–73.1	73.0	72.5–77.3	78.4	75.9–79.2	0.003	0.115	0.002	0.404
T2	8/13/11	72.5	70.8–79.1	75.4	73.5–79.0	78.8	75.3–83.8	0.052	–	-	-
T3	8/13/9	73.1	70.3–77.1	75.6	72.5–79.0	79.3	75.1–81.9	0.061	–	-	-
T4	10/12/10	73.4	71.0–76.6	75.6	73.2–80.3	77.9	76.0–80.6	0.039	0.582	0.032	0.517
wAS											
T1	10/13/11	13.9	3.6–44.3	8.8	3.4–30.4	43.9	8.0–88.2	0.076	–	-	-
T2	8/13/11	7.7	3.7–14.1	8.8	2.7–17.5	15.6	2.6–63.9	0.649	–	-	-
T3	8/13/9	9.0	5.4–17.7	9.4	2.5–15.0	10.5	5.2–46.2	0.477	–	-	-
T4	10/12/10	4.6	2.4–13.8	4.8	3.1–15.3	24.2	16.6–45.1	0.014	1.000	0.020	0.057
FS											
T1	10/13/11	0.218	0.209–0.227	0.200	0.194–0.21	0.216	0.196–0.228	0.042	0.056	1.000	0.190
T2	8/13/11	0.225	0.209–0.231	0.204	0.192–0.222	0.219	0.193–0.229	0.227	–	-	-
T3	8/13/9	0.202	0.193–0.218	0.205	0.188–0.230	0.204	0.193–0.214	0.99	–	-	-
T4	10/12/10	0.206	0.201–0.212	0.195	0.187–0.226	0.215	0.199–0.225	0.428	–	-	-

Of the preterm participants, 20 were born as nonsingletons and 14 as singletons. But in comparison, those did not differ statistically in the mean values of OCLR, CI, wAS, or FS.
